# High frequency of the X-chromosome inactivation in young female patients with high-grade glioma

**DOI:** 10.1186/1746-1596-8-101

**Published:** 2013-06-19

**Authors:** Gang Li, Zhiguo Zhang, Tianbo Jin, Hongjuan Liang, Yanyang Tu, Li Gong, Zhongping Chen, Guodong Gao

**Affiliations:** 1 Department of Neurosurgery, Tangdu hospital, the Fourth Military Medical University, 710038 Xi’an, China; 2 National Engineering Research Center for Miniaturized Detection Systems, School of Life Sciences, Northwest University, 710069 Xi’an, China; 3 Department of Clinical Experimental Surgery, Tangdu hospital, the Fourth Military Medical University, 710038 Xi’an, China; 4 Department of Pathology, Tangdu Hospital, the Fourth Military Medical University, 710038 Xi’an, China; 5 Department of Neurosurgery, State Key Laboratory of Oncology in Southern China, Cancer Center, Sun Yat-sen University, 510060 Guangzhou China

**Keywords:** Skewed X-chromosome inactivation, Androgen receptor gene, Glioma, High-grade, Cancer predisposition, Laser microdissection

## Abstract

**Background:**

Gliomas are common tumors and high-grade ones account for 62% of primary malignant brain tumors. Though current evidence have suggested that inherited risks play a role in glioma susceptibility, it was conveyed that glioma was such a complex disease, and the direct genetic contribution to glioma risk factors and its relation to other factors should be discussed more deeply. X-chromosome inactivation (XCI) is the mechanism by which gene dosage equivalence is achieved between female mammals with two X chromosomes and male mammals with a single X chromosome. As skewed XCI has been linked to development of some solid tumors, including ovarian, breast, and pulmonary and esophageal carcinomas, it is challenging to elucidate the relation of skewed XCI to high-grade gliomas development.

**Objective:**

The present study aimed to determine the general concordance between XCI pattern in blood cells and brain tissues, and SXCI frequencies in female patients with high-grade glioma compared to healthy controls.

**Methods:**

1,103 Chinese females without a detectable tumor and 173 female high-grade glioma patients, were detected in the study. Normal brain tissues surrounding the lesions in gliomas were obtained from 49 patients among the 173 ones, with the microdissection using a laser microdissection microscope Genomic DNA was extracted from the peripheral blood cells and the normal brain tissues from the subjects. Exon 1 of androgen receptor (*AR*) gene was amplified, and its products of different alleles were resolved on denaturing polyacrylamide gels and visualized after silver staining. The corrected ratios (CR) of the products before and after *Hpa*II digestion were calculated.

**Results:**

Occurrence of SXCI was detected in both the patients and controls at similar frequencies. However, the phenomenon, as defined as CR ≥ 3, was more frequent in the patients aging ≤40 (23.6%) compared to the corresponding reference group (5.1%, *P* <0.0001). When CR ≥ 10 was adopted, the frequencies were 5.5% and 1.6%, respectively. Their difference did not attain statistical significance (*P* = 0.10). When detected, both blood cells and brain tissue were compared after determination of a high concordance of XCI between blood cells and brain tissue collected from the same individuals (*n* = 48, r =0.57, *P* <0.01).

**Conclusions:**

The data from the current study demonstrated that SXCI may be a predisposing factor for development of high-grade glioma in young female patients and further study will verify its suitability as a biomarker to assess susceptibility of young female patients to high-grade glioma.

**Virtual slides:**

The virtual slide(s) for this article can be found here: http://www.diagnosticpathology.diagnomx.eu/vs/1935066233982578

## Background

The incidence of brain tumors is approximately 18.71 per 100,000 person-years (i.e., 11.52 per 100,000 for benign tumors and 7.19 per 100,000 person-years for malignant tumors) worldwide [1]. In comparison, in Shanghai core city, one of the most developed areas of China, in 2007 the overall age-specific incidence of primary central nervous system tumors was 80 per 100,000 person-years (i.e., 42 per 100,000 person-years for males, and 38 per 100,000 person-years for females, respectively) (unpublished data). Glioma is one such common malignancy, accounting for up to 30% of all brain tumors and 80% of the primary malignant brain tumors [2].

To date, the causes and mechanisms of gliomas remain to be elucidated, while hereditary genetic disorders such as neurofibromatoses (type 1 and type 2) and tuberous sclerosis complex are known to predispose to development of glioma [3]. However, glioma is a very complex disease and high-grade gliomas, including anaplastic gliomas and glioblastomas, account for the majority of gliomas, representing 62% of cases [4]. Thus, novel approaches are needed to identify susceptibility genes for association with glioma risk and the molecular mechanisms responsiable for glioma development [5]. Towards this end, Wrench *et al*. [6] in 2009 reported genome-wide screening data on association with glioma susceptibility and thereafter, a number of such association studies have been carried out for gliomas [5,7,8]. These explorations involved identification of susceptibility genes for glioma development and to date, eight glioma susceptibility loci have been identified by candidate gene-association studies [9]. Altered genes, gene promoters, and single-nucleotide polymorphisms (SNPs) could induce susceptibility to glioma development [10], while detection of gene mutations, deletions and translocations using DNA sequencing can identify genomic regions responsible for developing glioma [10]. A number of molecular alterations, such as *EGFR*, *TP53*, *RPA3*, *PTEN*, and *CDKN2A* have therefore been detected [11-13] and SNPs of some genes have also been identified in gliomas [5-8,14,15]. These studies have improved our knowledge and understanding of glioma development.

Our research is focused on skewing X-chromosome inactivation (SXCI). It is well known that in female mammals there are two X chromosomes, one of which is silenced epigenetically during early embryo development, thereby making female X-chromosome gene dosage largely equivalent to that of males [16-18], which occurs randomly [16]. Because of this random process, adult female tissues are cellular mosaics, wherein half of the cells contain an active maternal X chromosome (Xm) and the other half contain an active paternal X chromosome (Xp) [18]. This random moderate skewing might occur by chance due to the small number of stem cells undergoing X-inactivation during embryogenesis and results in a Gaussian distribution of X-chromosome inactivation ratios with a mean of 1 to 1. However, skewed inactivation ratios of ≥3 to 10 were infrequently detected in cord blood cells of neonates and, importantly, heritability of skewed X-chromosome inactivation patterns (XCIP) is a highly uncommon event [19,20]. Previous studies have shown that SXCI is associated with the development of breast [21-23], ovarian [24], lung [25] and esophageal cancers [26]. In glioma, an age-standardized incidence rate was higher among females than males [27]. Thus, this study investigated whether the imbalanced inactivation of X chromosomes in female somatic cells associates with an increased risk of glioma development.

## Subjects and methods

### Study population

A total of 173 female patients with glioma were recruited between November 2006 and December 2010 into our ongoing molecular epidemiological study in the Departments of Neurosurgery of Tangdu hospital and Xijing hospital, both of which are affiliated to The Fourth Military Medical University (FMMU) in Xi’an city, China. All glioma cases were without any previous history of other cancers and the patients had not undergone any prior chemotherapy or radiotherapy. There were no age, sex, or disease stage restrictions for case recruitment. All glioma tissue sections were re-evaluated by two pathologists according to WHO classifications and if there was discrepancy, they reviewed the tissue section together to sovle the differences by careful discussion. All of the 173 gliomas were high-grade gliomas, 85 of which were classified as anaplastic diffuse astrocytomas (WHO III), 29 as mixed anaplastic oligo-astrocytoma (WHO III), and 59 as primary glioblastomas (WHO IV) [28]. The median age of patients was 61 years (range between 14 and 73 years old). The clinicopathological features and the treatment strategies of all the patients are shown in Table 1.

**Table 1:** **Clinicopathological features of patients with glioma (*n*** **= 173)**

**Clinicopathological features**	**WHO III**		**WHO IV**
	anaplastic	mixed anaplastic	glioblastoma multiforme
	astrocytoma	oligo-astrocytoma	
**Total Cases**	85	29	59
**Mean Age (yrs.)**	64	59.5	53.6
**Kps**			
≥ 80	74	27	42
<80	11	2	17
**Surgery**			
Gross total resection	82	26	38
Partial resection	3	1	15
Biopsy	0	2	6
**Adjuvant treatment**			
Radiotherapy	50	11	38
Chemotherapy	5	5	15
Radiotherapy and Chemotherapy combination	30	13	6

Moreover, a random 1,103 healthy unrelated female individuals were recruited between June 2006 and August 2012 from the Medical Examination Center at Tangdu hospital. All of the chosen healthy subjects were Han Chinese living in Xi’an city and its surrounding areas. The median age was 55 years (range between 16 and 96 years old). A detailed recruitment and exclusion criteria was used, i.e., subjects with chronic diseases and conditions involving vital organs such as the heart, lung, liver, kidney and brain, and/or had severe endocrinological, metabolic or nutritional diseases were excluded from this study. The use of human tissues and blood samples in this study was approved by the Human Research Committee of the Fourth Military Medical University for Approval of Research Involving Human Subjects. A written informed consent was obtained from all the subjects or their custodians. All specimens were handled and made anonymous according to the ethical and legal standards.

### Demographic and clinical data

Demographic and personal data were collected through an in-person interview using a standardized epidemiological questionnaire, including age, sex, ethnicity, residential region, tobacco smoking, alcohol consumption, education levels, and family history of cancer. For patients, detailed clinical information was also collected through a medical chart review or consultation with treating physicians. Levels of plasma carcinoembryonic antigen and alpha-fetoprotein were detemrined in all control subjects to make sure that they were healthy and without any cancers.

### Blood samples collection, tissue laser microdissection, and DNA extraction

Peripheral blood was taken from the elbow vein or the head superficial vein, and treated immediately with an anticoagulant containing sodium citrate (22 g/L) and sodium chloride (8.5 g/L) from all 173 glioma patients and 1,103 healthy controls. The blood samples were then stored at −70°C until use.

**Figure 1:**
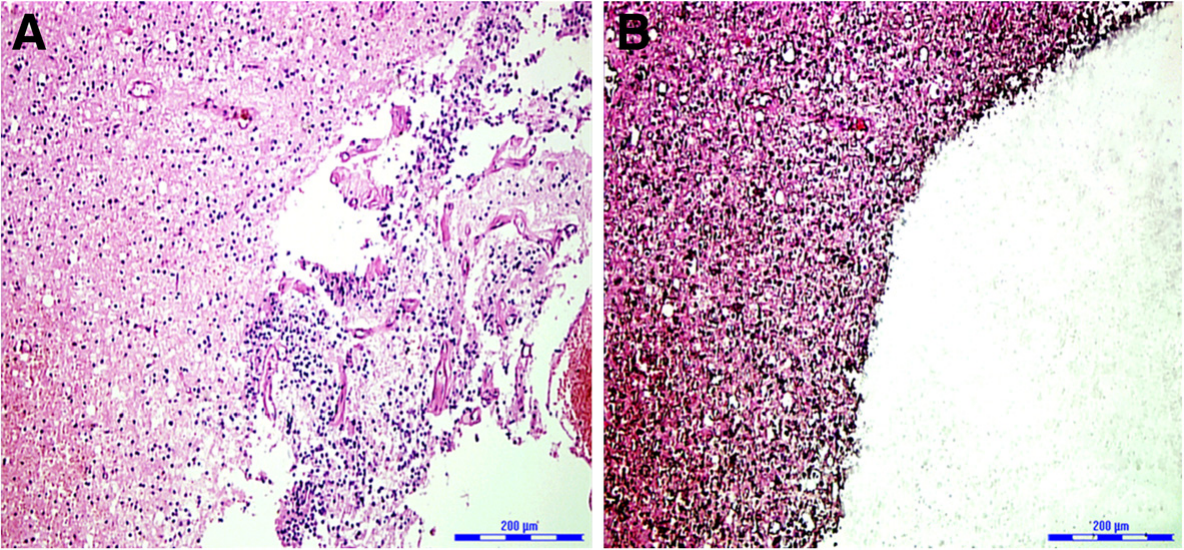
**Representative H&E stained brain tissue sections of the same individual diagnosed as mixed anaplastic oligoastrocytoma (AOA).** (**A**) Before laser microdissection and (**B**) after laser microdissection. ×100.

Tissue samples were also collected from the glioma patients, i.e., the same size (1.0 × 1.0 cm) of tissue was collected from both lesions and the surrounding normal brain. Ten 10-μm thick tissue sections (1.0 × 1.0 cm) prepared from the paraffin blocks were placed on a UV-absorbing membrane for microdissection using a laser microdissection microscope (LMD6000; Leica Microsystems, Wetzlar, Germany) as described previously [29,30]. Specifically, H & E stained tissue sections, both glioma and the surrounding normal brain tissue, were mounted on a microstat and dissected by an UV laser in a motorized optical beam scanning mode (Figure 1). The dissected cells fell by gravity into the cap of a microcentrifuge tube with a volume of 0.5 mL. The cap was filled with 40 μL lysate buffer (10% sodium dodecyl sulphate, 5 mmol/L sodium chloride, 1 mmol/L Tris–HCl and 0.5 mmol/L ethylenediaminetetraacetic acid) and 10 μL proteinase K (20 mg/mL). The microcentrifuge tubes were placed in a 48°C water bath and digested with proteinase K in a lysate buffer for 12–20 hours. Subsequently,,genomic DNA was extracted using a QIAamp Kit (Qiagen, Germany) according to the manufacturer’s instructions, and examined by 2% agarose electrophoresis and stored at −20°C. DNA concentration and purity was determined by an ultraviolet spectrophotometer (Eppendorf, Hamburg, Germany).

### Analysis of skewed X chromosome inactivation (SXCI)

The analysis is based on differential inactivation of X chromosomes of female somatic tissues and the CAG short-tandem repeat (STR) polymorphism at the *AR* gene exon 1 [31]. There are two *Hha*I and two *Hpa*II restriction sites at the locus 100 bp upstream to the CAG STR with a heterozygosity frequency of around 90% [31,32]. X-chromosome inactivation is associated with methylation of these restriction sites, i.e., if these sites are methylated, indicating the inactive X chromosome, this gene can not be transcribed, whereas if unmethylated, indicating the active X chromosome in females or male X chromosome, the gene can be transcribed [24].

Thus, we used this pricinple to digest DNA with methylation-sensitive endonucleases, followed by PCR with primers flanking these restriction sites and the highly polymorphic STR, to distinguish between the transcriptionally active and inactive X chromosome in heterozygous female subjects. In females with random X-chromosome inactivation, the amplification products from both alleles should be equal, with a ratio of approximately 1 to 1. In the neoplastic tissues, most of which originate from single cell clones [25,26], the ratio will change markedly compared with the surrounding normal tissues. It is worth noting that a remarkable deviation of the ratio has been observed in apparently non-neoplastic cell populations, such as peripheral blood cells from female subjects, which is defined as SXCI [33,34].

Genomic DNA (1 μg) was digested with 0.5 μL of *Hpa*II (10 U/μL; Promega, Madison, WI, USA) in 2 μL of 10 mol/L reaction buffer, 0.2 μL of 10 g/L bovine serum albumin and 7.3 μL of deionized water at 37°C for 4 hours. The reaction was then terminated by incubation of the sample mixture at room temperature for 30 minutes as suggested by the manufacturer. After that, a nested PCR was conducted as described previously [25,26]. A negative and water-blank control was included in each batch of PCR. The reaction fidelity of *Hpa*II digestion was guaranteed by parallel negative controls with the enzyme omitted from the reaction mixture. In addition, the whole assay was repeated twice.

After that, the amplification efficacy was demonstrated by electrophoresis of 2% agarose gels. The amplification products (4 μL of each) were mixed with the same volume of the loading buffer (1 g/L xylene cyanole, 1 g/L bromophenol blue, in formamide), loaded onto the 10% polyacrylamide gel containing 8 mol/L urea, resolved through electrophoresis with the Mini-VE system (Amersham Biosciences Corp., San Francisco, CA) at a voltage of 80 v for 8 hours, and then visualized after silver staining as described previously [32]. For the samples whose allelic differences at the CAG STR were small (one or two repeats), a longer gel (26-cm long and 0.75-mm thick) was used for the resolution with the SE660 system (Amersham). The results were recorded, and the intensities of the products from both alleles were analyzed by using an image-analyzing system (LabWorks 3.0, UVP, Cambridge, UK). To avoid the potential interference of possible preferential amplification of one of the alleles, we used the corrected ratio (CR) to evaluate the X-chromosome inactivation pattern by comparing the allelic difference of a sample before and after *Hpa*II digestion. CR was derived by dividing the ratio of the upper-band intensity to the lower-band intensity of the sample after digestion by that of the same sample before digestion. If CR was <1, the reciprocal value was considered. In the present study, CR ≥3, which indicated the expression of the same allele in more than 75% of the cells examined, was used to define SXCI. In addition, we also used CR ≥10 as a more stringent criterion for defining SXCI.

### Statistical analysis

Statistical analysis was performed using an SPSS package for Windows (Version 13.0; SPSS Inc., Chicago, IL, USA). The likelihood ratio test was used to determine the difference in SXCI frequency among various age groups. The χ^2^ test was used to compare categorical variables. Pearson’s correlation analysis was performed on XCI corrected ratio (CR) data obtained from the 49 glioma patients whose blood and brain tissues samples were accessible simultaneously. A *P* value of <0.05 (two-tailed) was considered statistically significant.

## Results

**Figure 2:**
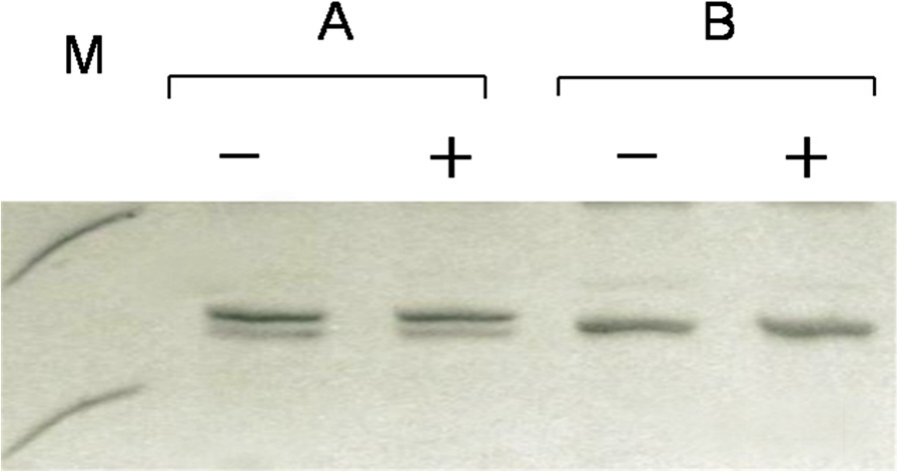
**Representative amplified and enzyme-digested AR exon 1 with (A) and without (B) CAG STR polymorphism.** +, *Hpa*II digestion; -, no *Hpa*II digestion; **M**, DNA marker, the upper and lower bands representing positions of 300 and 200 bp, respectively.

In this study, we amplified AR gene exon 1 in both blood and tissue samples to assess X-chromosome inactivation. Our data showed that among these 1,103 healthy female subjects, 1,001 (90.8%) were polymorphic at the CAG STR (Figure 2), indicating informative cases for X-chromosome inactivation. The ages of the informative cases ranged from 16 to 96 years old, with a median age of 55 years. Among the 173 female patients with glioma, 166 (96.0%) were shown to be polymorphic at the CAG STR. Their ages at diagnosis ranged from 14 to 73 years, with a median age of 61 years old. Statisically, there was no significant difference between the polymorphism frequencies for the cancer patient and the control groups (96.0% *vs* 90.8%, *P* > 0.05). If combined, the frequency of CAG STR polymorphism was 91.5% (1,167/1,276) which is similar to the data published previously [22-26].

**Figure 3:**
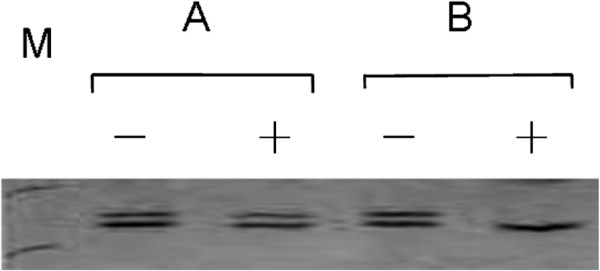
**Representative X-chromosomal inactivation patterns. A**, SXCI CR = 5.5, >3 and **B**, CR = 29, >10 . **M**, DNA marker, with the upper and lower bands at positions of 300 and 200 bp, respectively.

We used a cut-off point of CR ≥3 or CR ≥10 to describe the X-chromosomal inactivation skewing in these subjects (Figure 3). SXCI frequeny was evaluated for patients with gliomas, and compared to that of the reference group (Table 2). Specifcially, using CR ≥3 as cut-off point, the SXCI frequencies were 19.3% and 16.5%, respectively in patients and controls, respectively, whereas using CR ≥10 as the cut off point, the SXCI frequencies were 4.8% and 3.0%, respectively. The differences were not statistically significant (*P* > 0.05). Since previous studies have confirmed the link of SXCI to aging, the age factor should be taken into consideration in the current study. The SXCI frequency (using CR ≥3) in patients with age of ≤ 20 years (28.6%) was found to be higher than that of healthy controls (4.5%) (*P* = 0.00015). When CR ≥ 10 was adopted, the frequencies were 5.7% and 2.3%, respectively, and their difference did not attain statistical significance (*P* = 0.28). Interestingly, the SXCI frequency in patients with age of ≤40 years old (23.6%) was found to be much higher than that of the healthy controls (5.1%) with SXCI defined as CR ≥3 (*P* < 0.0001). However, when CR ≥ 10 was adopted, the frequencies were 5.5% and 1.6%, respectively, and their difference did not attain statistical significance (*P* = 0.10). However, in the patients and references who were more than 40 years old, excessive skewing, as defined by CR ≥ 3 and CR ≥ 10, were observed in 17.1% and 21.7% and 4.5%, 3.6% of the subjects, respectively. There was no statistically significant difference between these two groups (*P* > 0.05). We then associated blood cell SXCI with clinical stages of gliomas and found that using CR ≥3, the SXCI frequencies in WHO grades III and IV gliomas were 20% (11/55) and 18.9% (21/111), respectively. There was no significant difference between them (*P* > 0.05).

**Table 2:** Skewed X-chromosomal inactivation frequencies in glioma patients of various age groups and the corresponding controls

**Groups**	**Age range (median: ys)**	**Numbers examined**	**Numbers with CR ≥ 3(%)**	***P*-value**	**Numbers with CR ≥ 10(%)**	***P-*** **value**
Patients	14-20 (17)	35	10 (28.6)	0.00015	2 (5.7)	0.28
Controls	16-20 (18)	133	6 (4.5)		3 (2.3)	
Patients	14-40 (19)	55	13 (23.6)	<0.0001	3 (5.5)	0.10
Controls	16-40 (24)	312	16 (5.1)		5 (1.6)	
Patients	41-73 (57)	111	19 (17.1)	0.28	5 (4.5)	0.59
Controls	41-96 (67)	689	149 (21.7)		25 (3.6)	
Patients	14-73 (61)	166	32 (19.3)	0.37	8 (4.8)	0.22
Controls	16-96 (55)	1001	165 (16.5)		30 (3.0)	

**Figure 4:**
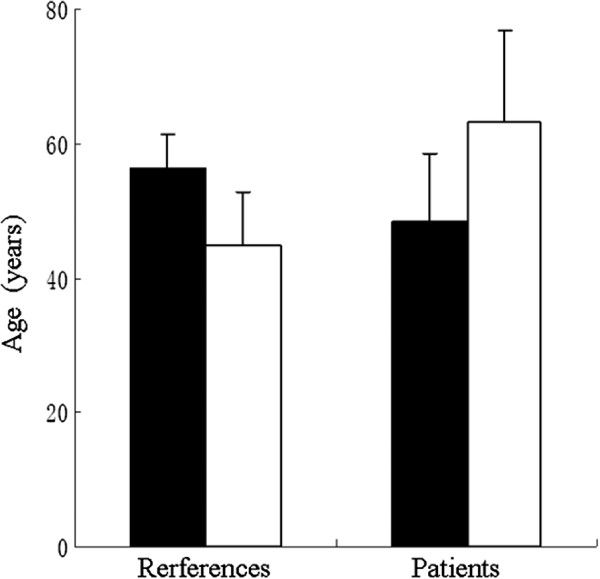
**Average ages of glioma patients and controls with SXCI (CR ≥ 3, filled bar) and without SXCI (empty bar).** The standard deviations denoted on each bars. The differences in both control and patients are statistically significant (*p* < 0.05 and 0.005, respectively).

Furthermore, we also determined the average ages of the subjects with and without SXCI for both groups (Figure 4). Using CR ≥3, the average age of the healthy female subjects with SXCI was more than 11 years older than that of those without SXCI (*P* < 0.05). In contrast, the average age of the cancer patients with SXCI was more than 16 years younger than those without SXCI (*P* < 0.01; Figure 4).

**Figure 5:**
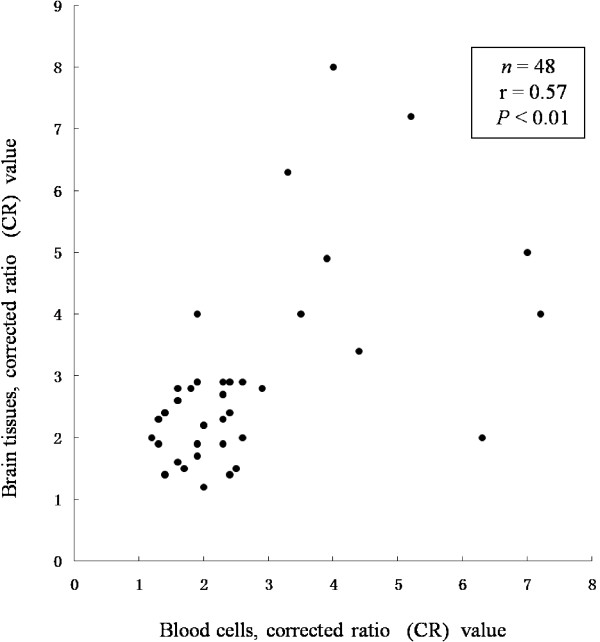
Scatterplot of corrected ratio (CR) values of SXCI between blood cells and normal brain tissues of the same individual.

In addition, we also compared SXCI in blood cells with brain tissues in 49 patients and found that both blood cells and brain tissues of the 48 cases (98.0%) were polymorphic at the CAG STR, informative cases for X-chromosome inactivation. Both blood cells and brain tissue from the same individuals showed a high concordance of XCI (*n* = 48, r = 0.57, *P* <0.01) (Figure 5). These data suggest that the detected females have a relatively high level of concordance in XCI pattern between hematopoietic and CNS brain tissues.

The relationship between SXCI in blood cells and clinical stages of the cancer was also assessed. With the criterion of CR ≥ 3 adopted, the frequencies for the cases of WHO grades III and IV were 20% (11/55) and 18.9% (21/111), respectively. There was no significant difference among them (*P* > 0.05).

## Discussion

In the current study, we extracted genomic DNA from the peripheral blood or brain tissue samples from 1,103 Chinese female control subjects and 173 female patients with high-grade glioma. We then amplified androgen receptor (*AR*) exon 1 and digested it with *Hpa*II to assess X-chromosome inactivation. We found that similar SXCI frequencies occurred in both patients and controls. However, SXCI (with an adopted CR ≥ 3) frequency was 23.6% in patients with an age of 40 years or younger compared to the healthy controls (5.1%). Moreover, SXCI frequencies showed a high concordance of XCI between blood cells and brain tissues. These data demonstrated that SXCI was a predisposing factor for development of high-grade glioma in young female patients.

Despite recent advancement in therapy for glioma, such as surgery, radiotherapy, photodynamic therapy, and chemotherapy, the clinical outcome of patients with high-grade glioma remains poor, especially in patients with WHO IV glioma (9.8% 5-year survival rate) [4]. Thus, it is held widely that early detection of glioma could prolong the survival of glioma patients, and some detected biomarks have been verified their suitability to assist significantly in the evaluation of biological activity in gliomas and even have prognostic value [35,36]. Furthermore, some correlated studies have shown that inherited risk factors do play a significant role in glioma susceptibility, and that a heritable component of glioma had a twofold elevated risk in individuals with a positive family history [37,38], and rare genetic syndromes increased the risk of glioma [39]. GWAS studies have identified common susceptibility variants at 5p15.33 (*TERT*), 8q24.21 (*CCDC26*), 9q21.3(*CDKN2A*-*CDKN2B*), 20q13.33(*RTEL1*) and 11q23.3 (*PHLDB1*) [5-8]. These fingings could help us to understand the altered genes and their expressions which are involved in the development of glioma. Meanwhile other studies, including our current study, have investigated gene SNPs or chromosome levels of alterations as a means of predicting genetic susceptibility for glioma development. Our current data showed that SXCI occurred more frequently in glioma patients with an age of 40 years or younger compared to the healthy controls, which could be further evaluated as a biomarker for glioma susceptibility in young patients.

A number of studies have reported on the association between XCI skewing and developmental disorders in females [21-26]. These studies usually examined XCI patterns in blood samples because tissues of the organ of interest (e.g., CNS) were either unavailable or difficult to obtain. Thus, in the current study, we compared SXCI frequencies between blood cells and the brain tissues and found that SXCI frequencies had a high concordance between blood cells and brain tissues. This finding suggests that blood is a useful surrogate brain tissue for such an analysis.

Previous studies showed that SXCI occurrence in blood cells was associated with autoimmune diseases [40-43] and linked to the development of certain female cancers [44]. For example, in 1999, Buller *et al*. reported that patients with invasive ovarian cancer had an increased frequency of SXCI compared to those without ovarian tumor [24]. In a study by Kristiansen *et al*., SXCI (CR ≥ 10) frequency was shown to be markedly increased in young patients (≤ 45 years old) with breast cancer (13%) compared to that of the control group (1%) [22]. A similar phenomenon was also observed in familial breast cancer patients without a detectable *BRCA*-1 or *BRCA*-2 mutation [23], lung cancer [25] and esophageal carcinomas [26].

In the current study, SXCI (CR ≥ 3) frequency was determined to be as high as 23.6% in young patients (≤40 years) with gliomas, which was significantly higher than that in the corresponding control subjects (5.1%). Moreover, the average age at diagnosis in the cancer patients with SXCI was 11 years younger than that in patients without SXCI. However, SXCI was first described in 1987 during a clonality assay based on *AR* gene polymorphism in tissue samples [45]. To date, the mechanism of SXCI is largely unknown and was believed to be due to selection for, or against, alleles on the active X chromosome. Such selection may depend upon gene expression of the gene or the interaction with other genes [44]. SXCI may also occur when the size of the pool of the embryonic precursor cells undergoing X-chromosome inactivation is too small to avoid stochastic variation [45]. Moreover, SXCI may be attributable to relatively small selective advantages, such as X-chromosome rearrangements and mutations in X-linked genes [45,46].

In addition, our current study did address some aspects in this field of study. For example, the previous studies raised a question whether the XCI pattern in different tissues could be predicted by testing haematopoietic cells. Our current study showed a general concordance of XCI pattern between blood cells and brain tissues. Moreover, it is known that population admixture may cause type-I error (false positive) for association studies. In our study, all the samples we used were from the same hospital to avoid two or more definite selection bias which is why they did not differ in geographical distributions or genotype frequencies. The race of all participants was limited to Han Chinese who lived in Xi’an city or nearby areas; thus, substantial population admixture can be ignored in our study. In addition, it has long been recognized from a theroretical perspective that an additional explanational for SXCI ratios might include mutations in the X-inactivation process itself, which causes one chromosome to be chosen over another at the time of X inactivation in the early embryo [47,48]. Mutations of the X-inactivation pathway genes are thought to be rare, but studies of individuals with such mutations may provide more information regarding the regulation of the X-inactivation pathway.

## Conclusion

In this study, we observed an excellent concordance of SXCI frequency between blood cells and brain tissues. SXCI is a predisposing factor for the development of high-grade glioma in young female patients. However, further study is needed to verify whether SXCI is useful as a biomarker for prediction of glioma development in young female patients.

## Competing interests

The authors declare that there is no competing interest.

## Authors’ contributions

ZC and GG designed the study. GL and TJ participated in study design and experiment coordination, performed the molecular genetic evaluation, and drafted the manuscript. ZZ and LG performed the statistical analysis and joined to draft the manuscript. YT, HL and GL followed-up all patients and helped to improve the manuscript. All authors have read and approved the final manuscript.
